# Predictive Role of F_2_-Isoprostanes as Biomarkers for Brain Damage after Neonatal Surgery

**DOI:** 10.1155/2017/2728103

**Published:** 2017-10-08

**Authors:** L. J. Stolwijk, P. M. A. Lemmers, M. Y. A. van Herwaarden, D. C. van der Zee, F. van Bel, F. Groenendaal, M. L. Tataranno, M. Calderisi, M. Longini, F. Bazzini, M. J. N. L. Benders, G. Buonocore

**Affiliations:** ^1^Department of Neonatology, University Medical Center Utrecht, Utrecht, Netherlands; ^2^Brain Center Rudolf Magnus, University Medical Center Utrecht, Utrecht, Netherlands; ^3^Department of Pediatric Surgery, University Medical Center Utrecht, Utrecht, Netherlands; ^4^Department of Pediatrics, Obstetrics and Reproductive Medicine, University of Siena, Siena, Italy

## Abstract

**Objective:**

Neonates have a high risk of oxidative stress during anesthetic procedures. The predictive role of oxidative stress biomarkers on the occurrence of brain injury in the perioperative period has not been reported before.

**Methods:**

A prospective cohort study of patients requiring major surgery in the neonatal period was conducted. Biomarker levels of nonprotein-bound iron (NPBI) in plasma and F_2_-isoprostane in plasma and urine before and after surgical intervention were determined. Brain injury was assessed using postoperative MRI.

**Results:**

In total, 61 neonates were included, median gestational age at 39 weeks (range 31–42) and weight at 3000 grams (1400–4400). Mild to moderate brain lesions were found in 66%. Logistic regression analysis showed a significant difference between plasma NPBI in patients with nonparenchymal injury versus no brain injury: 1.34 umol/L was identified as correlation threshold for nonparenchymal injury (sensitivity 67%, specificity 91%). In the multivariable analysis, correcting for GA, no other significant relation was found with the oxidative stress biomarkers and risk factors.

**Conclusion:**

Oxidative stress seems to occur during anaesthesia in this cohort of neonates. Plasma nonprotein-bound iron showed to be associated with nonparenchymal injury after surgery, with values of 1.34 umol/L or higher. Risk factors should be elucidated in a more homogeneous patient group.

## 1. Introduction

The impact of surgery and anaesthesia on the young infants' brain is a subject of ongoing debate. Major surgery has been shown to give a higher risk of death or neurodevelopmental impairment in a large, retrospective cohort study in very low-birth-weight infants [1]. Commonly used inhalational anaesthetics are reported to be neurotoxic in experimental studies and induce neuronal apoptosis [2, 3]. Studies on the clinical effect of anaesthetics on the developing brain are challenging. Infants with major noncardiac congenital anomalies requiring neonatal surgery (esophageal atresia, intestinal atresia, anorectal malformation, and gastroschisis) have an increased risk of a neurodevelopmental delay [4]. These patients are at risk of oxidative stress due to the anaesthetic procedure including administration of sevoflurane, the fraction of inspired oxygen, and pain, especially since the tendency is to keep them highly saturated during surgery [5]. Fluctuations in blood pressure, arterial CO_2_, and duration of anaesthesia pose a risk for the neonatal brain in terms of developing brain injury [6]. In 63% of patients with noncardiac congenital anomalies in this cohort study, brain lesions were visible on their postoperative MRI [7]. The exact timing of the brain injury may help to discover the pathogenesis of these lesions. In this process, biomarkers of oxidative stress might provide insight into aetiology and pathogenic factors. To date, very few reported on the role of the anaesthesic procedure in this patient group. In this study, we hypothesize that biomarkers of oxidative stress (i.e., plasma and urinary F_2_-isoprostane and plasma nonprotein-bound iron) are associated with brain injury and aim to clarify the aspects of oxidative stress during anaesthesia. The association between perioperative parameters, such as mean arterial blood pressure, arterial CO_2_, administration of opioids and duration of anaesthesia, and biomarkers for neuronal injury, was investigated.

## 2. Material and Methods

### 2.1. Patients

This prospective, cohort study was performed from January 2014 to December 2015 at the Neonatal Intensive Care Unit of the Wilhelmina Children's Hospital Utrecht, Utrecht, the Netherlands. All eligible newborns with noncardiac congenital anomalies, requiring major neonatal surgery, were enrolled. This study was approved by the Medical Ethical Committee of the University Medical Center Utrecht. Parents were asked for written informed consent, in accordance to the principles of the Declaration of Helsinki (64th WMA General Assembly, Fortaleza, Brazil, October 2013).

Exclusion criteria consisted of critical cardiac congenital malformations, major congenital anomalies of the central nervous system, and insufficient Dutch language proficiency of the parents.

## 3. Methods

### 3.1. Biomarkers

**Figure 1:**
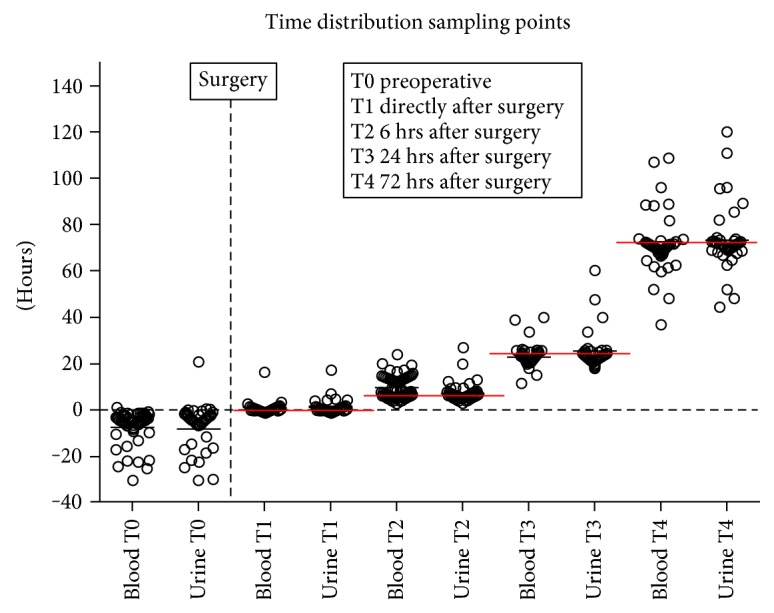
Time distribution sampling points.

Heparinized blood samples of 1 ml were drawn from the indwelling peripheral arterial catheter and inserted for clinical purposes. These samples were centrifuged immediately, to obtain platelet-poor plasma, and butylated hydroxytoluene (BHT) 1% *w*/*v* in methanol (5 *μ*l per ml of plasma) was added to prevent the *in vitro* lipid peroxidation [8]. Urine samples of 4 ml were collected from already inserted urinary catheters or noninvasively from a gauze placed in the infants' diaper. Six time points were chosen: within 24 hours prior to surgery; immediately after surgery; and 6, 24, and 72 hours after surgery for measurement of biomarkers of neuronal injury (Figure 1). Blood and urine samples were stored in a refrigerator at −80°C until analysis. Plasma levels of NPBI were detected by high-performance liquid chromatography (HPLC) as described by Paffetti et al. [9] using an HPLC system consisted of quaternary pumps, vacuum degassers, thermostated autosampler, DAD detector, and fluorimeter detector (Agilent 1100 series). The method is based on preferential chelation of NPBI by a large excess of the low-affinity ligand of nitrilotriacetic acid (NTA).

Determination of F_2_-isoprostanes in plasma and urine was described by Casetta et al. [10]. The method was centered around an API 4000 Tandem Mass Spectrometer (AB Sciex, Toronto, Canada) equipped with an electrospray ionization (ESI) probe on the Turbo-V source. The chromatographic configuration was an Agilent 1200 stack. The chromatographic configuration was an Agilent 1200 stack, which included a binary pump, a thermostated well-plate autosampler (kept at 8°C), and a column oven.

The chromatography separation was carried out on a Dionex Acclaim C18-120 3um 2 × 100 mm, maintained at 30°C, and flowed at a rate of 300 *μ*l/min by a mixture of an aqueous solution of acetic acid at 0.3% (eluent A) and acetonitrile (eluent B) according to the following gradient program. Upon injection, the eluent composition was maintained at 25% of B for 1′ and then moves up to 90% in 4′. Subsequently, the concentration of eluent B reaches 100% for 1′. The equilibration step was performed at 25% of B for 4′. Total chromatographic run was 10 minutes.

For measurements, the tandem mass spectrometer has been run in multiple reaction monitoring (MRM) with the electrospray source operating in negative ion mode and by exploiting the transition m/z 353.3 > 193.2 for F_2_-isoprostanes and 357.3 > 197.2 for the isotopically-labeled form used as internal standard (d4–8-iso PGF_2*α*_, Cayman Chemical Co., Ann Arbor, MI, USA) [11].

### 3.2. Neuroimaging

A postoperative MRI was performed on a 3.0 Tesla whole-body Achieva system (Philips Medical Systems, Best, the Netherlands) as part of routine clinical care. The scanning protocol included T1-, T2-, diffusion, and susceptibility weighted images.

### 3.3. Data

Obstetric and neonatal data, perioperative data, as well as details on anaesthetic and surgical management were collected from patient charts.

### 3.4. Statistical Analysis

Statistical procedures were performed using IBM SPSS statistics software package (IBM^®^ SPSS Statistics version 20, IBM Corp. Armonk, NY, USA, and R statistical computing) [12]. Data are presented as mean ± standard deviation (SD) or as median and range when indicated. Comparison of biomarker levels before and after surgery was performed using the Wilcoxon signed-rank test and the Bonferroni post hoc correction for multiple testing. The Mann– Whitney *U* test was performed to compare biomarker levels and presence of brain injury. A multivariable regression analysis was performed using the cumulative concentration of biomarker levels in the first 60 hours after surgery. Parameters investigated were gender, type of congenital anomaly, endoscopic procedure, and the occurrence of parenchymal injury, after correcting for gestational age. Receiver operating characteristic curves (ROC) were calculated at different time points from T1 to T4, in order to detect the best time point correlating with damage.

## 4. Results

Of the 84 patients admitted to the NICU between January 2014 and December 2015 and who underwent major surgery in the neonatal period, parents of 73 neonates were approached and asked for consent of their infant to participate in the study. Exclusion criteria consisted of emergency surgery (*n* = 4), absence of one of the parents (*n* = 3), insufficient Dutch language proficiency (*n* = 3), or diagnosis made during surgery (*n* = 1). In 61 of these 73 neonates, parental informed consent was given. Clinical data are presented in Table 1 (Supplemental Table 1 available online at https://doi.org/10.1155/2017/2728103 and Supplemental Table 2. Anesthetics during surgery). Figure 1 shows the time distribution of each sampling point.

### 4.1. Oxidative Stress Biomarkers

**Figure 2:**
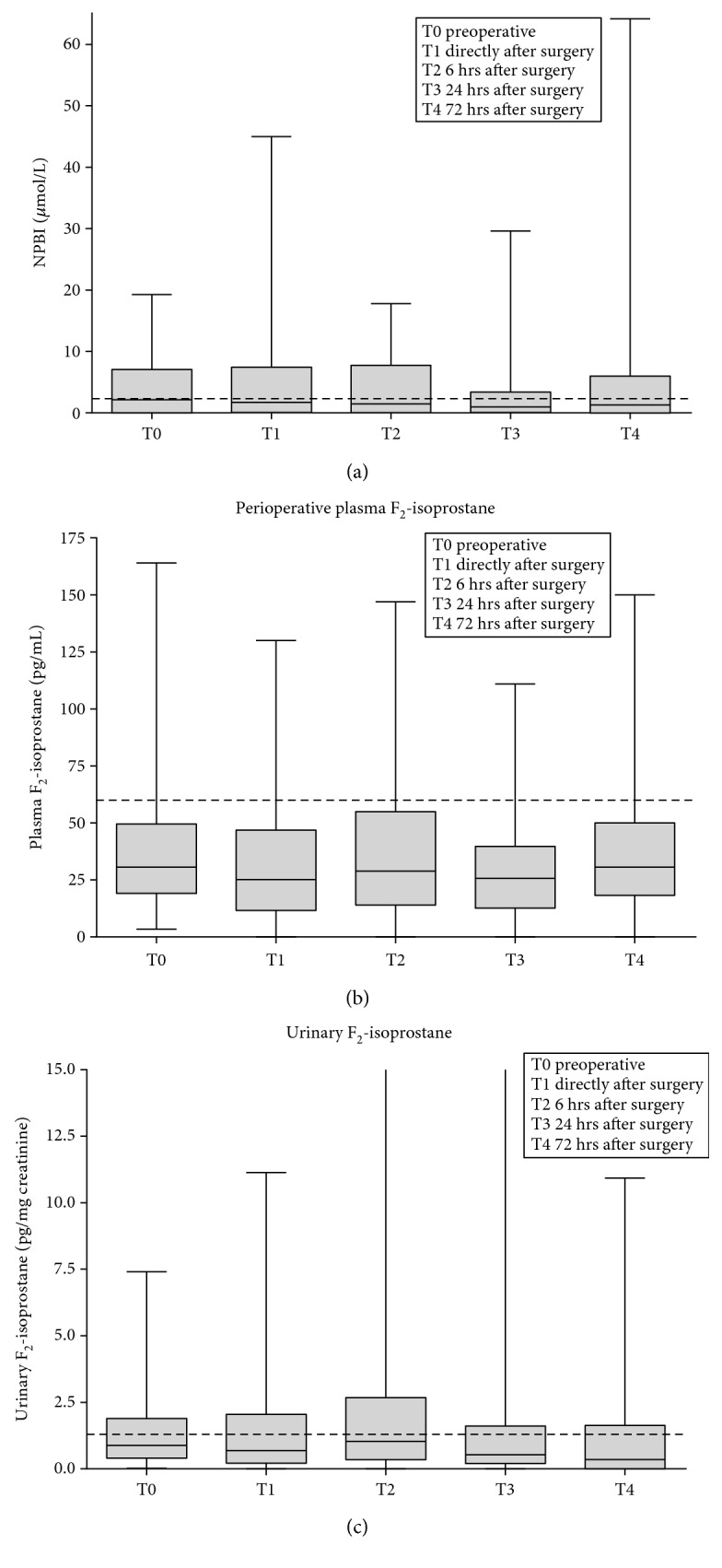
Perioperative data of oxidative stress biomarkers. (a) Data of plasma NPBI at each time point. Dotted line indicates 2.3 umol/L NPBI, and normal values of plasma NPBI are below this cutoff value. (b) Data of plasma F_2_-isoprostane at each time point. Dotted line indicates 60 pg/mL F_2_-isoprostane, and normal values of plasma F_2_-isoprostane are below this cutoff value. (c) Data of urinary F_2_-isoprostane at each time point. Dotted line indicates 1.3 pg/mg creatinine F_2_-isoprostane, and normal values of urinary F_2_-isoprostane are below this cutoff value.

Plasma and urinary levels of F_2_-isoprostane and plasma NPBI were not significantly different before surgery in comparison to values after surgery (T0 versus T1–T4, Wilcoxon signed-rank test with post hoc Bonferroni, *p* > 0.3) (Figure 2) (Supplemental Table 2).

### 4.2. Brain Lesions

**Figure 3:**
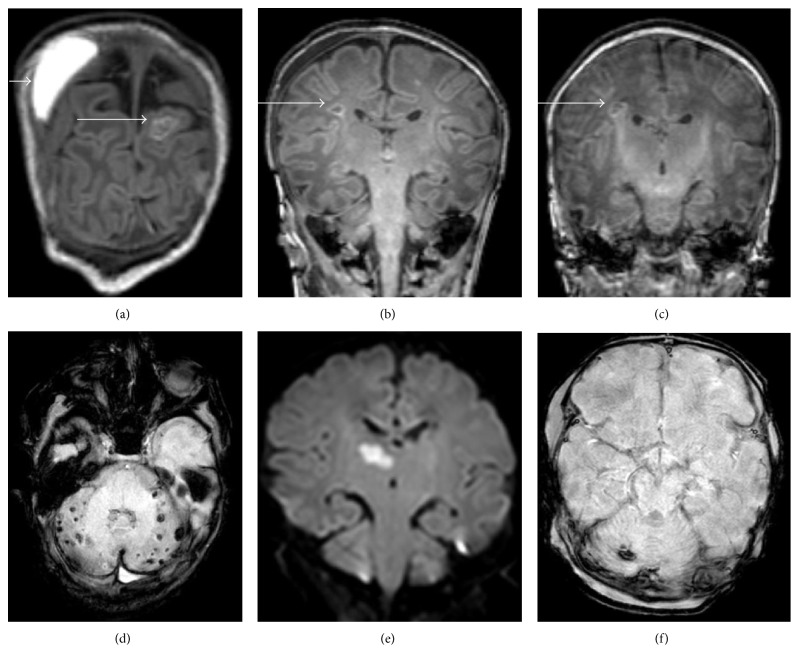
Examples of parenchymal and nonparenchymal brain injury. (a) Coronal T1-weighted image: cortical infarction and subdural haemorrhage; (b) coronal T1-weighted image: white matter lesion; (c) coronal T1-weighted image: white matter lesion; (d) susceptibility weighted image: multiple punctate cerebellar lesions; (e) diffusion-weighted image: thalamic infarction; (f) susceptibility weighted image: cerebellar haemorrhage.

**Figure 4:**
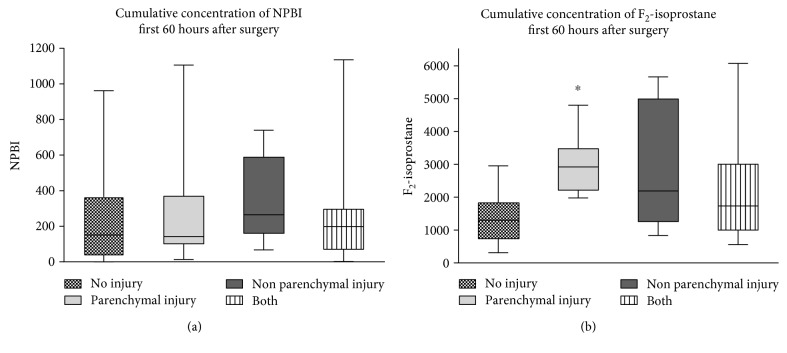
A significant difference in plasma F_2_-isoprostane between “no injury” and “parenchymal injury” was found using the Mann–Whitney *U* test with post hoc Bonferroni correction (*U* = 11.0, *p* < 0.01, and *r* = −0.70). ^∗^Significant difference between parenchymal injury and no injury.

Parenchymal and/or nonparenchymal brain lesions were found in 66% of 58 postoperative MRI scans (Table 2 and Figure 3). In 18 neonates, a combination of parenchymal and nonparenchymal injury was visible. In 51% of the neonates, parenchymal injury was present and 55% of these lesions were visible on the diffusion weighted images (DWI), which indicates timing of the injury to be in the perioperative period. The cumulative concentration of plasma F_2_-isoprostane of patients with no brain injury showed significantly lower levels than patients with parenchymal injury (Mann–Whitney *U* test, *U* = 11.0, *p* < 0.01, *r* = −0.70, Figure 4), indicating higher concentrations of F_2_-isoprostane in patients with brain injury.

### 4.3. Multivariable Linear Regression

In the multivariable analysis, using the cumulative concentration starting directly after surgery to 60 hours after surgery for each biomarker, correcting for GA, none of the potentially influencing factors showed a significant linear relation with plasma and urinary F_2_-isoprostane or plasma NPBI. Parameters investigated consisted of gender, type of congenital anomaly, endoscopic procedure, and the occurrence of parenchymal injury (Figure 4). This indicates that after correcting for gestational age, the absence or presence of brain injury did not influence the overall concentrations of the oxidative stress biomarkers in the perioperative period.

### 4.4. ROC Curve Analysis

**Figure 5:**
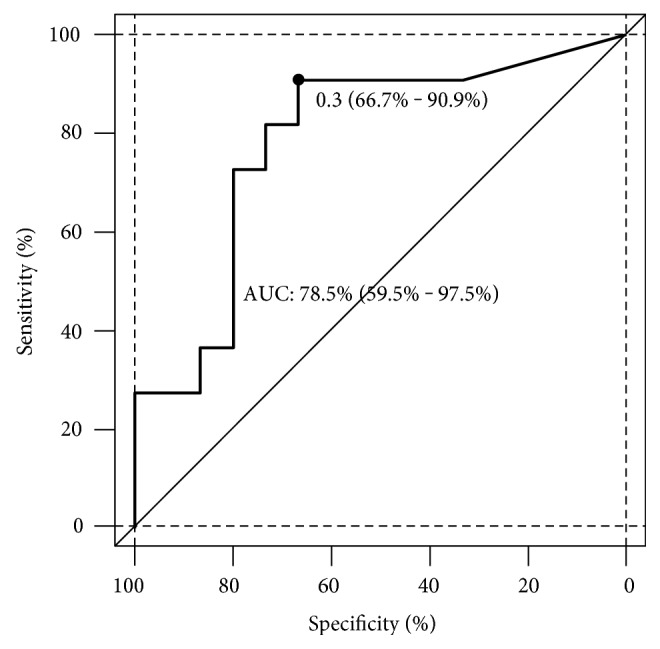
Receiver operating characteristic (ROC) curve analysis for NPBI at 72 hours after surgery. The area under the curve indicates that NPBI at 72 hours after surgery allows to differentiate neonates with nonparenchymal injury from no injury. The area under the curve was 0.785 (95% confidence interval: 0.595–0.975), with 66.7% specificity and 90.9% sensitivity. 1.34 *μ*M/l or higher was identified as predictive threshold to have nonparenchymal injury.

A logistic regression was performed for each time point. A significant difference in plasma NPBI was found between patients with nonparenchymal injury (78.5%) and patients with no brain injury after anaesthesia. The results indicate plasma NPBI at 72 hours after surgery as the best early predictor for nonparenchymal injury. The determination of NPBI levels at 72 hours after surgery allows in differentiating neonates having nonparenchymal brain injury from no brain injury: AUC 78.5% (95% confidence interval: 0.595–0.975), with 66.7% specificity and 90.9% sensitivity (Figure 5). The threshold 1.34 μM/l was identified as predictive value for having nonparenchymal injury. A predictive threshold value at one specific time point could not be identified for F_2_-isoprostane.

## 5. Discussion

This study investigated oxidative stress biomarkers in neonates undergoing neonatal surgery for noncardiac congenital anomalies. In this cohort, 66% of the neonates had mild to moderate brain lesions visible on their postoperative MRI. A combination of parenchymal and nonparenchymal injury was found in 30% of the infants. In the total patient cohort, after correcting for gestational age, perioperative biomarker concentrations of F_2_-isoprostane and NPBI were not correlated to perioperative brain injury. In the ROC curve analysis, assessing the subgroups no brain injury, parenchymal and nonparenchymal brain injury at each time point, and oxidative stress seems to occur as a consequence of anaesthesia. This was shown by elevated levels of NPBI in plasma and urinary F_2_-isoprostane after surgery in patients with parenchymal injury in comparison to patients with no brain injury. In addition to that, the level of 1.34 umol/L plasma NPBI was identified as risk threshold for nonparenchymal injury. Despite the bias related to heterogeneity, this study points out that these neonates are at risk of oxidative stress during the anaesthetic procedure.

There is no previous literature available on biomarkers in patients with noncardiac congenital anomalies requiring surgery. This could be explained by the fact that patients with congenital malformations are structurally excluded from trials. However, biomarkers of neuronal injury might be of great value in this vulnerable group of patients.

The hypothesis was to find an increase in biomarker values after surgery in comparison to preoperative values. The basal level of oxidative stress before surgery was reported for the first time. Interestingly, the values of F_2_-isoprostane at baseline—before surgery—were low, in comparison to the findings of Comporti et al. Newborns in this study had values between 50–150 pg/ml after birth, and blood samples were obtained from the umbilical vein. Our results show a median F_2_-isoprostane of 30 pg/ml creatinine at baseline, which is drawn from the arterial catheter at a median age of two days. Apparently, the oxidative stress at birth caused by the transition from a low oxygen pressure in utero to a relatively high oxygen pressure after birth was resolved [19].

Oxidative stress is a unifying term for the end product of several diseases, which can be produced by free radicals. Biomarkers are defined as indicators of normal processes or measures of pathological processes [13]. Free radicals damage the endothelial cell and cause inflammatory reactions and brain cell damage, which can be evaluated by an increased level of nonprotein-bound iron. NPBI has been proven to be a predictive biomarker of neonatal brain damage in preterm infants [14]. This biomarker seems to play a pivotal role in identifying neonates at risk of brain damage. F_2_-isoprostanes, discovered by Morrow et al, are a product of free radical-induced injury by peroxidation of lipids. This biomarker is formed via nonenzymatic peroxidation of polyunsaturated fatty acids mediated by free radical production [13]. This peroxidation of arachidonic acid is produced by a noncyclooxygenase mechanism [15] and is a noninvasive method to monitor lipid peroxidation in vivo. An increase of F_2_-isoprostane in plasma and urine occurs after hypoxic-ischaemic brain injury and reperfusion [16] and predicts the risk of having brain injury. Previous studies have shown that F_2_-isoprostane is a reliable and chemically stable biomarker [17, 18]. Importantly, it is previously described that plasma F_2_-isoprostane levels are inversely correlated with gestational age [19]. This is also the case in healthy infants, Friel et al. found an increased level of F_2_-isoprostane in a cohort of 12 infants at the age of one month [5]. Furthermore, lipid exposure to high concentrations of NPBI also leads to the formation of isoprostanes.

Reactive oxygen species (ROS) are known to cause oxidative stress in the newborn infant, leading to damage to cell structures like lipids and membranes [5, 20, 21]. Free radical oxidative damage in the newborn is involved in diseases like retinopathy of prematurity, bronchopulmonary dysplasia, necrotizing enterocolitis, and patent ductus arteriosus [21]. In addition, the developing brain of the neonate, with its high concentration of polyunsaturated fatty acids, is highly susceptible to hypoxia and hyperoxia [22]. These pathological circumstances cause oxidative stress reactions, in particular in the neonate with their immature antioxidant defenses [23]. White matter is selectively injured, since the developing oligodendrocyte is the main target of oxidative stress in hypoxia-ischaemia and systemic inflammation [22, 24]. Brain injury has previously been described in the group of patients with cardiac congenital anomalies undergoing major surgery [25]. Our results show a high incidence of mild to moderate brain lesions in infants with noncardiac congenital anomalies as well.

The sensitivity of the rapidly developing brain of the neonate undergoing major surgery to brain injury is threefold. First, the developing brain tissue is sensitive to free radicals. ROS cause oxidative stress [20], in particular, in newborns with their reduced enzymatic and nonenzymatic antioxidant defense [21]. Neonates undergoing surgery are exposed to fluctuating fractions of inspired oxygen. During induction of anaesthesia, an increased supply of oxygen is administered, to prevent hypoxia during intubation, which enhances the risk of free oxygen radicals. Secondly, inhalational anaesthetics are thought to cause neurotoxic effects in the developing newborn brain [2, 26, 27]. The anaesthetic causes an increase in apoptosis, an impaired neurogenesis, and neuroinflammation in animal experimental studies [28]. Third, cerebral perfusion is at risk due to immature cerebral autoregulatory ability in preterm infants or a loss of cerebral autoregulation caused by sevoflurane anaesthesia [29]. Presence or absence of autoregulatory ability can be determined by blood pressure and cerebral oxygenation, measured by near-infrared spectroscopy [30]. In case of pressure-passive perfusion, fluctuations in respiratory and cardiovascular parameters pose a risk for cerebral saturation and perfusion. Maintenance of cerebral blood flow is critical to ensure adequate oxygenation of the brain.

This study has several limitations. First, with the use of F_2_-isoprostane only, a specific type of brain damage was investigated, involving the prostaglandin metabolism. The second limitation is that our cohort consists of a heterogeneous patient group. In order to solve this problem, each patient is used as their own control, with the baseline measurement before surgery. Furthermore, the bias consisted of differences in gestational age, weight, different surgical techniques, and differences in administered dose of anaesthesia and pain medication. The strength of this study, however, is that it is the first study to investigate these biomarkers in neonates with noncardiac congenital anomalies, at multiple time points, in combination with the use of MRI. Also, the clinical use of these biomarkers is investigated in this prospective study where cerebral monitoring is applied in the perioperative period as well. The combination of biomarkers and MRI offers the opportunity to identify neonates at risk of brain injury more precisely, which might be of great value to develop tailored therapy and preventive measures for brain injury in the future.

## 6. Conclusion

Despite the bias related to heterogeneity of the study group, our results showed a risk of oxidative stress during anaesthesia in neonates.

## Supplementary Material

Supplemental Table 1. Overview of congenital anomalies. Supplemental table 2. Statistics of Wilcoxon Signed Rank test.

## Figures and Tables

**Table 1:** Clinical data.

	*n* = 61
Gestational age (weeks)	38.9 (30.9–41.6)
Male, *n* (%)	36 (59%)
Birth weight (grams)	3000 (1405–4430)
Birth weight *z*-score	−0.25 (−2.1–1.9)
Small for gestational age, *n* (%)	2 (3%)
Preterm, *n* (%)	15 (25%)
Apgar score 1 minute	9 (2–10)
Apgar score 5 minutes	10 (2–10)
Postnatal age in days at time of surgery	2 (0–8)
Postnatal age in hours at time of surgery	39.9 (2–184)
*Surgery*	
Thoracoscopy, *n* (%)	18 (30%)
Laparoscopy, *n* (%)	16 (26%)
Laparotomy, *n* (%)	23 (38%)
Duration surgery (minutes)	115 (23–475)
Duration anaesthesia (minutes)	189 (63–563)
*Medication during anaesthesia*	
Sevoflurane, *n* (%)	60 (98%)
Isoflurane, *n* (%)	1 (2%)
Sufentanil, *n* (%)	60 (98%)
Propofol, *n* (%)	14 (23%)
Morphine, *n* (%)	17 (28%)
Caudal analgesia, *n* (%)	13 (21%)
Suxamethonium, *n* (%)	1 (2%)
Atracurium, *n* (%)	51 (84%)
Rocuronium, *n* (%)	9 (15%)

Data displayed in median (range) or indicated otherwise.

**Table 2:** Incidence of brain injury.

Brain injury	*n* ^∗^
No injury	20
Parenchymal injury	13
Nonparenchymal injury	7
Parenchymal and nonparenchymal injury	18

^∗^MRI was not available in three patients: one was declined by the parents, one had a preoperative MRI scan only, and one patient was diagnosed with Down's syndrome.

## Abbreviations

NCCA: Noncardiac congenital anomalies
NPBI: Nonprotein bound iron
NICU: Neonatal intensive care unit
NIRS: Near-infrared spectroscopy
GA: Gestational age.
