# Supplementary material for: Predictive Role of F2-Isoprostanes as Biomarkers for Brain Damage after Neonatal Surgery
Source: Dis Markers. 2017 Oct 8;2017:2728103. doi: 10.1155/2017/2728103 (PMC5651108; doi:10.1155/2017/2728103)
Supplement: Supplementary file 1 — Supplemental Table 1. Overview of congenital anomalies. Supplemental table 2. Statistics of Wilcoxon Signed Rank test. [file 2728103.f1.docx]

**Supplemental Table 1. Overview of congenital anomalies**

| **Non-cardiac congenital anomaly** | **n** |
| --- | --- |
| Esophageal atresia | 18 |
| Intestinal atresia | 14 |
| Gastroschisis | 11 |
| Anorectal malformation | 5 |
| Intestinal malrotation / volvulus | 4 |
| Urogenital malformation | 4 |
| Other | 5 |
