# Supplementary material for: Predictive Role of F2-Isoprostanes as Biomarkers for Brain Damage after Neonatal Surgery
Source: Dis Markers. 2017 Oct 8;2017:2728103. doi: 10.1155/2017/2728103 (PMC5651108; doi:10.1155/2017/2728103)
Supplement: Supplementary file 2 [file 2728103.f2.docx]

**Supplemental table 2. Statistics of Wilcoxon Signed Rank test**

|  | **Control** | **Versus** | **T^#^** | **p** | **r** |
| --- | --- | --- | --- | --- | --- |
| **Plasma F_2_-isoprostane** | T0 | T1 | 419 | 0,375 | -0,09053 |
|  | T0 | T2 | 411 | 0,801 | -0,02638 |
|  | T0 | T3 | 343 | 0,115 | -0,16169 |
|  | T0 | T4 | 422 | 0,912 | -0,01135 |
| **Urinary F_2_-isoprostane** | T0 | T1 | 309 | 0,372 | -0,09619 |
|  | T0 | T2 | 256 | 0,478 | -0,07701 |
|  | T0 | T3 | 271 | 0,097 | -0,17412 |
|  | T0 | T4 | 219 | 0,112* | -0,23031 |
| **Plasma NPBI** | T0 | T1 | 308 | 0,909 | -0,0118 |
|  | T0 | T2 | 290 | 0,35 | -0,09748 |
|  | T0 | T3 | 238 | 0,136 | -0,15318 |
|  | T0 | T4 | 319 | 0,826 | -0,02281 |

*^#^Wilcoxon Signed Rank test, *post hoc Bonferroni correction was applied. Time points are indicated by: T0: preoperative, T1: directly after surgery, T2: 6 hours after surgery, T3: 24 hours after surgery, T4: 72 hours after surgery.*
